# Phenolic Esters of *O*-Desmethylvenlafaxine with Improved Oral Bioavailability and Brain Uptake

**DOI:** 10.3390/molecules181214920

**Published:** 2013-12-04

**Authors:** Yang Zhang, Yan Yang, Sen Zhao, Zhichao Yang, Hong Yang, J. Paul Fawcett, Youxin Li, Jingkai Gu, Tiemin Sun

**Affiliations:** 1 Key Laboratory of Structure-Based Drug Design and Discovery, Shenyang Pharmaceutical University, Ministry of Education, Shenyang 110016, China; 2 Research Center for Drug Metabolism, College of Life Sciences, Jilin University, Changchun 130021, China; 3 School of Chemical and Pharmaceutical Engineering, Jilin Institute of Chemical Technology, Jilin 132022, China; 4 School of Pharmacy, University of Otago, PO Box 56, Dunedin 9054, New Zealand

**Keywords:** *O*-desmethylvenlafaxine, phenolic ester, prodrug, pharmacokinetics, brain uptake, rat, dog

## Abstract

*O*-Desmethylvenlafaxine (desvenlafaxine, ODV) is a recently approved antidepressant which in some clinical studies failed to meet a satisfactory end-point. The aim of this study was to prepare a series of phenolic esters of ODV and evaluate their potential as ODV prodrugs with improved brain uptake. Fifteen phenolic esters (compounds **1a**–**o)** were synthesized and their pharmacokinetic profiles evaluated in rat. The four compounds producing the highest relative bioavailability of ODV in rat (compounds **1c**, **1e**, **1n**, **1o**) were then studied to evaluate their brain uptake. Of these four compounds, compound **1n** (the piperonylic acid ester of ODV) demonstrated the highest C_max_ of ODV both in the rat hypothalamus and total brain. Finally the pharmacokinetics of **1n** were evaluated in beagle dog where the increase in relative bioavailability of ODV was found to be as great as in rat. This high relative bioavailability of ODV coupled with its good brain penetration make **1n** the most promising candidate for development as an ODV prodrug.

## 1. Introduction

Depression is a common mental disease estimated to affect some 350 million people worldwide [1]. In fact, a World Mental Health Survey conducted in 17 countries in 2012 found that about 5% of people reported having an episode of depression in the previous year [2]. The condition displays a high rate of lifetime incidence, early age onset, high chronicity and significant role impairment. Despite a wide range of pharmacotherapeutic options, response to antidepressant medication is subject to delayed onset and is highly variable. It is also not without significant adverse effects. Thus the search for improved antidepressant drugs remains an ongoing concern.

Venlafaxine (VEN) is a bicyclic phenylethylamine-based antidepressant which selectively blocks presynaptic reuptake of norepinephrine (NE) and serotonin (5-HT) without blocking histaminergic, muscarinic or α_1_-adrenergic receptors [3,4]. Because of this action as a selective 5-HT-NE reuptake inhibitor (SNRI), VEN has a wide therapeutic index and improved tolerability profile when compared with tricyclic antidepressants (TCAs) [5]. Apart from depression, VEN is also used to treat generalized anxiety, obsessive-compulsive and panic disorder as well as social phobia [6]. Its most common side effects are nausea, somnolence, dizziness, dry mouth, and sweating [7].

**Scheme 1:**
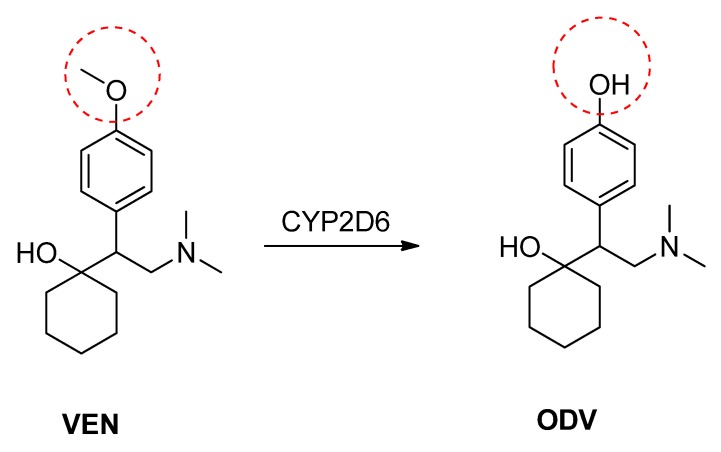
The conversion of venlafaxine to its principal metabolite *O*-desmethylvenlafaxine by CYP2D6.

In humans, VEN is well absorbed and subject to extensive first-pass metabolism in the liver by cytochrome P450 2D6 (CYP2D6). This makes it susceptible to CYP2D6 polymorphism and the associated variability in pharmacokinetics, efficacy and/or tolerability [8,9,10]. *O*-Desmethylvenlafaxine (ODV, also known as desvenlafaxine) is the major (56%) metabolite of VEN (Scheme 1) with antidepressant activity similar to that of VEN but with a much longer half-life [11,12,13]. ODV is mainly metabolized by UDP-glucuronosyltransferase enzymes [14], making it less prone to polymorphic pharmacokinetic and pharmacodynamic variability than VEN [10].

These favorable properties led to ODV being approved by the United States Food and Drug Administration in 2008 for the treatment of major depressive disorder but, interestingly, it was not approved in the European Union because of an unfavorable risk-benefit balance in both hot flushes and depression [15]. More recent studies have shown that the minimum effective dose of ODV (as the succinate salt) is 50 mg/day which may explain the lack of efficacy found in some studies [16,17]. ODV has been shown to have high bioavailability in humans (ca. 80%), raising the question of whether its somewhat unconvincing antidepressant efficacy is the result of low brain exposure after oral dosing. On this basis, we decided to compare the relative bioavailability and brain uptake of ODV with some of its phenolic esters as potential prodrugs of ODV.

Prodrugs are usually designed to improve passive and/or transporter-mediated intestinal absorption [18,19] to increase oral bioavailability [20,21,22] and/or tissue-selective delivery [23]. In some cases they are also used to reduce the side effects of a drug, particularly if it is to be used chronically or for chemotherapy [24,25]. In our previous work [26], a preclinical pharmacological evaluation of the *p*-methoxybenzoic acid ester of ODV (TP1) in rat was reported. It was shown that after oral administration, TP1 rapidly penetrates the rat brain and hypothalamus, is converted into ODV and demonstrates higher relative bioavailability than ODV. This proof-of-concept that lipophilic phenolic esters of ODV improve the oral bioavailability and brain uptake of ODV led us to synthesize a series of lipophilic phenolic esters of ODV (including TP1, compound **1k**) and examine their bioavailability and, in some cases, brain uptake in rat.

## 2. Results and Discussion

### 2.1. Chemistry

**Scheme 2:**
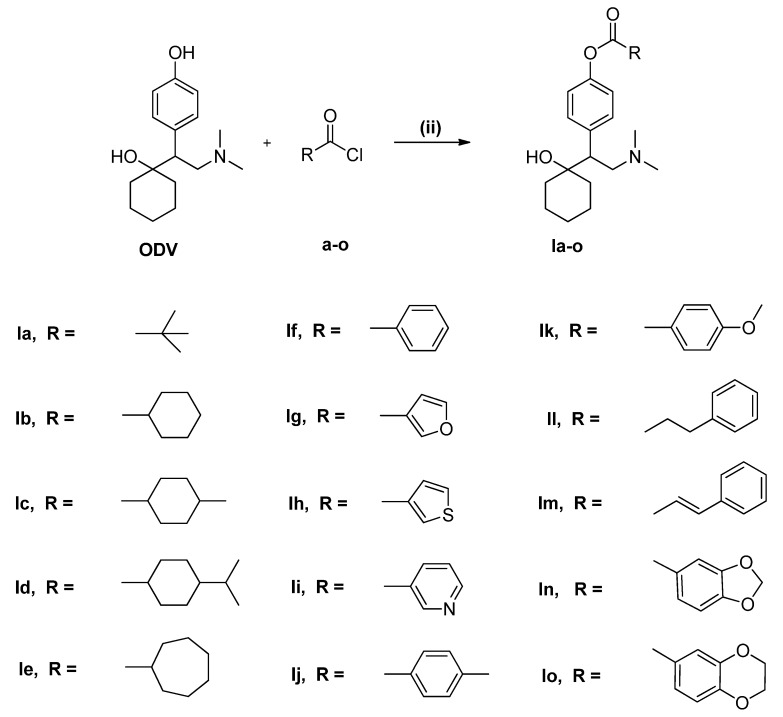
Formation of phenolic esters of ODV **1a**–**o** by reaction with RCOCl in anhydrous pyridine/THF at 0 °C for 1 h, followed by room temperature for 5 h.

Acyl chlorides **a**–**o** were synthesized from the corresponding carboxylic acids by reaction with SOCl_2_ in pyridine at 90 °C for 3 h [27]. Compounds **1a**–**o** were synthesized by treatment of ODV with the corresponding acyl chloride in the presence of anhydrous pyridine and THF at 0–25 °C for 6 h (Scheme 2). The target compounds were obtained in low to moderate yield [28].

### 2.2. Pharmacokinetic (PK) Studies in Rat

Compounds **1a**–**o** and ODV were administered orally to Wistar rats at a dose of 0.02 mmol/kg. Plasma samples were collected over 24 h and analyzed for ODV by liquid chromatography-tandem mass spectrometry (LC-MS/MS) [8,29,30]. Calculated log *P* (Clog *P*) values of compounds **1a**–**o** and ODV were predicted by Molinspiration. PK parameters including area under the plasma concentration-time curve (AUC), mean residence time (MRT), elimination half life (t_1/2_), peak concentration (C_max_) and time to reach C_max_ (T_max_) were calculated by non-compartmental analysis using DAS Version 3.0. The relative bioavailability of ODV (F) of compounds **1a**–**o** compared to ODV (as 100) was calculated as F = AUC_0-t_(**1a**–**o**)/AUC_0-t_(ODV) × 100%. Clog *P* values of the esters along with PK parameters of ODV produced from them are shown in Table 1.

**Table 1:** Clog *P* of the esters and PK parameters of ODV after oral administration of **1a**–**o** and ODV to rats at a dose of 0.02 mmol/kg (data are means ± SD, n = 4).

Compd.	Clog *P*	C_max_	T_max_	t_1/2_	AUC_0-t_	AUC_0-∞_	MRT	F
		μg/L	h	h	μg/L × h	μg/L × h	h	%
ODV	3.3	66.6 ± 14.6	1.25 ± 1.06	4.14 ± 1.21	219 ± 56	222 ± 59	6.03 ± 2.82	100
1a	4.8	7.67 ± 1.21	1.50 ± 0.71	2.13 ± 0.83	41.1 ± 3.8	41.1 ± 3.7	4.70 ± 0.47	18.7
1b	5.4	18.2 ± 3.4	1.50 ± 0.24	3.95 ± 1.84	152 ± 15	156 ± 20	6.99 ± 1.40	69.5
1c	5.2	98.8 ± 14.5	0.50 ± 0.12	5.47 ± 1.50	278 ± 86	293 ± 103	4.81 ± 2.06	127
1d	6.7	33.3 ± 18.0	0.92 ± 0.83	6.40 ± 5.30	222 ± 114	246 ± 148	6.31 ± 0.44	101
1e	5.9	118 ± 16	0.33 ± 0.04	2.39 ± 0.15	321 ± 83	321 ± 93	4.37 ± 1.39	146
1f	5.6	47.0 ± 13.4	2.00 ± 1.41	4.61 ± 2.28	216 ± 89	220 ± 87	4.53 ± 1.96	98.4
1g	4.2	9.36 ± 3.60	0.58 ± 0.59	1.65 ± 0.11	29.4 ± 0.2	29.4 ± 0.2	4.14 ± 0.10	13.4
1h	4.9	15.7 ± 4.6	0.50 ± 0.07	2.53 ± 1.65	58.7 ± 23.9	58.9 ± 23.6	4.92 ± 1.14	26.8
1i	4.0	10.5 ± 2.7	0.50 ± 0.12	1.19 ± 0.80	21.3 ± 2.8	21.3 ± 2.8	2.35 ± 0.40	9.73
1j	6.1	101 ± 31	0.42 ± 0.09	2.49 ± 0.44	241 ± 61	251 ± 80	2.77 ± 0.80	110
1k	5.7	96.3 ± 24.7	1.75 ± 0.35	4.32 ± 0.82	228 ± 38	231 ± 47	4.95 ± 1.85	104
1l	5.4	12.4 ± 13.5	1.50 ± 0.71	8.61 ± 3.09	27.8 ± 23.6	33.9 ± 23.7	9.29 ± 3.43	12.7
1m	5.4	30.4 ± 5.9	1.25 ± 0.35	6.35 ± 4.09	178 ± 60	197 ± 84	6.75 ± 1.60	81.3
1n	5.5	141 ± 48	1.00 ± 0.58	8.29 ± 5.12	421 ± 146	456 ± 153	4.42 ± 0.74	192
1o	5.1	87.7 ± 13.3	0.44 ± 0.10	9.02 ± 1.74	278 ± 85	318 ± 84	6.83 ± 0.73	127

**Figure 1:**
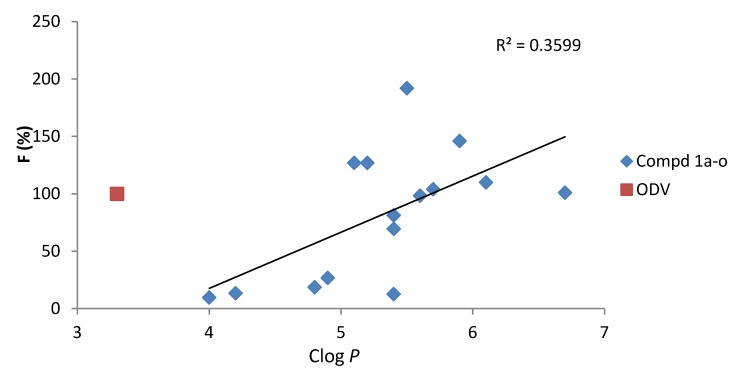
Relationship between F and Clog *P* values for compounds **1a**–**o** showing the linear regression line of best fit.

The results show that the Clog *P* values of all ODV esters are higher than that of ODV and span the range 4.0–6.7. They also show that the t_1/2_ values of ODV vary considerably and are approximately proportional to F, indicating that F is controlled by absorption of the esters. A plot of Clog *P vs.* F supports this in that it gives a reasonably linear correlation (r^2^ = 0.3599, Figure 1) with four compounds lying above the linear regression line and one below it. The presence of these outliers suggests that the rate of hydrolysis of the esters may also contribute to the F value. This is supported by the fact that the ester with R = –C(CH_3_)_3_ (compound **1a**) has a much lower F than ODV.

**Figure 2:**
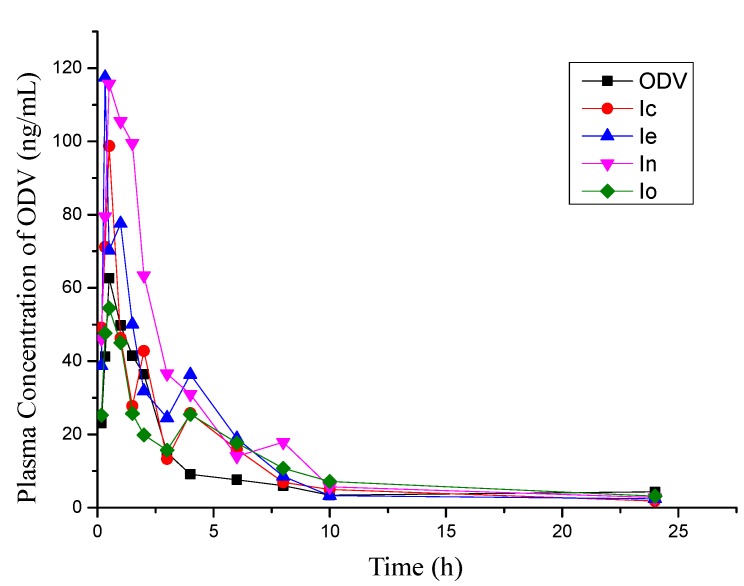
Plasma concentration-time profiles of ODV after administration of single oral doses (0.02 mmol/kg) of the four phenolic esters of ODV producing the highest relative bioavailability of ODV in rat (data are means, n = 4).

Considering the F values in relation to the structures of the esters, those containing an alicyclic ring (compounds **1b**–**e**) have F values that do not follow the trend of their respective Clog *P* values in that **1c** and **1e** are higher than ODV, whereas **1b** is lower. The compound with a phenyl group (compound **1f**) has a similar F to ODV but, for compounds with aromatic bioisosteres (compounds **1g**–**i**), F is much lower, consistent with their lower lipophilicity. Compounds with substituted phenyl groups (compounds **1j**, **1k**, **1n**, **1o**) have higher F values than ODV, especially compound **1n**. However, compounds with aralkyl groups (compounds **1l**, **1m**) have lower F values, particularly **1l**. On the basis of these results, the four compounds with the highest F values (*ie*., compounds **1c**, **1e**, **1n**, and **1o)** were selected for evaluation of their brain penetration. The mean plasma concentration-time curves of ODV after oral administration of these selected compounds and ODV are shown in Figure 2.

### 2.3. Brain Uptake Studies in Rat

**Figure 3:**
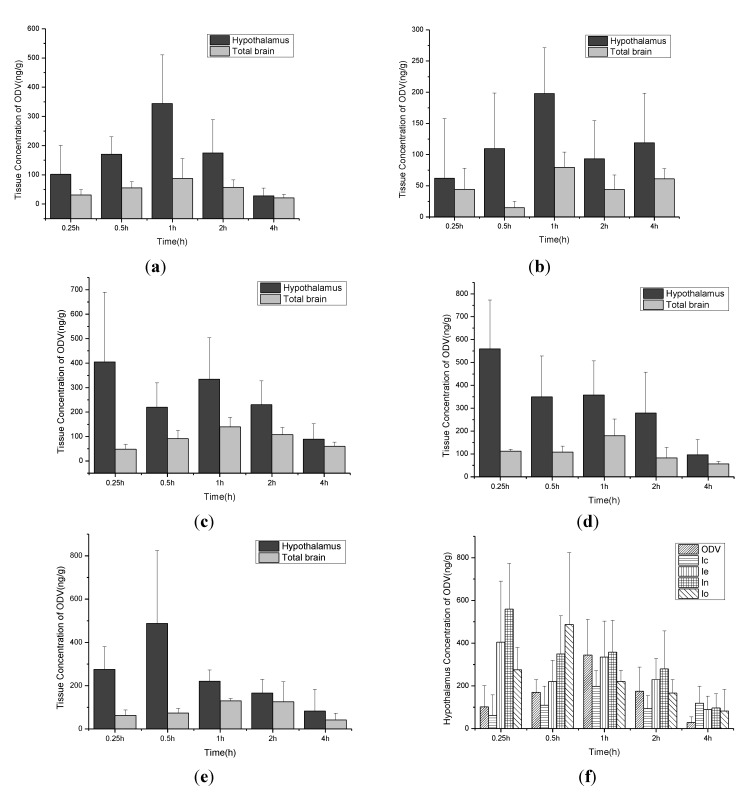
Concentrations of ODV in the hypothalamus and total brain produced after single oral doses of (**a**) ODV, (**b**) compound **1c**, (**c**) compound **1e**, (**d**) compound **1n** and (**e**) compound **1o**. (**f**) shows the concentrations of ODV in the hypothalamus after administration of ODV, **1c**, **1e**, **1n** and **1o** to rats at a dose of 0.06 mmol/kg (data are means + SD, n = 6).

For brain uptake studies, the selected compounds were administered to groups of Wistar rats at a dose of 0.06 mmol/kg [10,26]. Since the density of receptors for 5-HT and NE reuptake is high in the hypothalamus [31], the concentrations of ODV were determined in both hypothalamus and total brain where total brain represents the brain tissue after removal of the hypothalamus. The results, shown in Figure 3, indicate that the piperonylic acid ester of ODV (compound **1n)** produced the highest level of ODV in the hypothalamus at 0.25 h (514 ng/g) and the highest level in total brain at 1 h (180 ng/g). At 4 h, concentrations of ODV produced by all esters were higher than those produced by ODV itself.

### 2.4. PK Studies of **1n** and ODV in Beagle Dog

**Figure 4:**
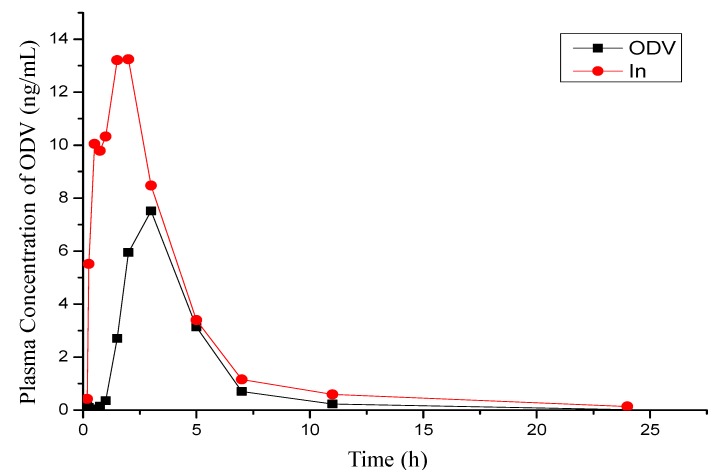
Mean plasma concentration-time profiles of ODV in a cross-over study involving oral administration of compound **1n** and ODV at a dose of 0.007 mmol/kg to a group of beagle dogs (data are means, n = 6).

A group of beagle dogs was administered single oral doses (0.007 mmol/kg) of compound **1n** and ODV in a cross-over study with a washout period of one week. As shown in Table 2 and Figure 4, **1n** was rapidly absorbed and metabolized to ODV in beagle dogs. Thus the T_max_ of ODV after administration of **1n** was much shorter than after administration of ODV (1.21 *vs.* 2.67 h), the C_max_ was much higher (20.8 *vs.* 7.55 µg/L) and the F value was nearly doubled, consistent with the results observed in rat.

**Table 2:** PK parameters of ODV in a cross-over study involving oral administration of compound **1n** and ODV at a dose of 0.007 mmol/kg to a group of beagle dogs (data are means ± SD, n = 6).

Compd.	C_max_	T_max_	t_1/2_	AUC_0–t_	AUC_0–∞_	MRT	F
	μg/L	h	h	μg/L × h	μg/L × h	h	%
ODV	7.55 ± 4.34	2.67 ± 0.58	1.55 ± 0.29	27.7 ± 12.1	27.8 ± 12.6	4.10 ± 0.70	100
**1n**	20.8 ± 6.6	1.21 ± 0.61	3.41 ± 1.54	55.1 ± 17.2	55.5 ± 16.9	3.91 ± 0.76	199

## 3. Experimental

### 3.1. General

ODV was purchased from Dingjin Chemical Co., Ltd (Zibo City, China). Other commercial reagents and solvents were used without further purification. Melting points were determined on a Walden Precision Apparatus Electrothermal 9300 apparatus and are uncorrected. ^1^H-NMR (400 MHz) and ^13^C-NMR (100 MHz) spectra were recorded on a Bruker Advance 400 nuclear magnetic resonance spectrometer. High resolution mass spectroscopy (HRMS) was carried out using a Triple-TOF 5600 mass spectrometer equipped with an electrospray ionization (ESI) source. Compound purity was assessed by HPLC analysis on an Agilent 1200 HPLC system and shown to be >95% in all cases. Animal experiments were carried out in compliance with the Guidelines for the Care and Use of Laboratory Animals of the National Research Council of USA, 1996 and related ethical regulations of Jilin University. Drug concentrations in the PK studies were determined by LC-MS/MS using an Agilent 1100 Series HPLC system and a QTRAP 2000TM mass spectrometer.

### 3.2. General Procedure for Synthesis of Compounds **a**–**o**

Compounds **a**–**o** were prepared from the corresponding carboxylic acids (9.2 mmol) and thionyl chloride (2 mL) with heating under reflux for 3 h. After removal of the thionyl chloride *in vacuo*, the resulting acyl chlorides were used without further purification.

### 3.3. General Procedure for Synthesis of Compounds **1a**–**o**

ODV (2.0 g, 7.6 mmol) was dissolved in anhydrous pyridine (40 mL) at ambient temperature. The appropriate acyl chloride in anhydrous THF (5 mL) was slowly added dropwise at 0 °C and the reaction mixture left with stirring at 0 °C for 1 h under nitrogen, followed by 5 h at room temperature. The reaction mixture was then added to water (100 mL) with vigorous stirring over 5 min, after which the pH of the reaction mixture was adjusted to 9.0 using 1N NaOH solution to precipitate the crude product. After standing overnight, the precipitate was filtered, washed with water and purified by column chromatography (silica gel, methanol-dichloromethane 1:15 v/v then 1:10 v/v) to give the desired products **1a**–**o**.

*4-(2-(Dimethylamino)-1-(1-hydroxycyclohexyl)ethyl)phenyl pivalate* (**1a**). ODV was treated with pivaloyl chloride (**a**, 0.9 g) to give **1a** (1.3 g, 48.1%) as a white powder; mp 131–133 °C; ^1^H-NMR (DMSO-*d_6_*) δ 0.91–1.57 (m, 10H, cyclohexane-CH_2_-), 1.29 (s, 9H, -C(CH_3_)_3_), 2.11 (s, 6H, -N(CH_3_)_2_), 2.84 (m, 2H, -CH_2_-N), 3.30 (m, 1H, Ar-CH), 6.95 (d, *J* = 8.4 Hz, 2H, Ar-H), 7.23 (d, *J* = 8.4 Hz, 2H, Ar-H); ^13^C-NMR (DMSO-*d_6_*) δ 21.22, 21.28, 25.55, 26.71 (3C), 33.43, 36.82, 38.45, 45.27 (2C), 51.95, 59.95, 71.99, 120.40 (2C), 130.14 (2C), 139.24, 148.85, 176.28; HRMS(ESI): *m/z* calcd. for [M+H]^+^: 348.2494; found: 348.2535.

*4-(2-(Dimethylamino)-1-(1-hydroxycyclohexyl)ethyl)phenyl cyclohexanecarboxylate* (**1b**). ODV was treated with cyclohexanecarbonyl chloride (**b**, 1.1 g) to give **1b** (1.5 g, 52.0%) as a white powder; mp 122–124 °C; ^1^H-NMR (DMSO-*d_6_*) δ 1.08–1.74 (m, 20H, 2 × cyclohexane-CH_2_-), 1.96 (m, 1H, CHCOO-), 2.11 (s, 6H, -N(CH_3_)_2_), 2.56 (m, 1H, -CH_2_-N), 2.84 (m, 1H, -CH_2_-N), 3.30 (m, 1H, Ar-CH), 4.99 (br, 1H, -OH), 6.95 (d, *J* = 8.2 Hz, Ar-H), 7.22 (d, *J* = 8.2 Hz, 2H, Ar-H); ^13^C-NMR (DMSO-*d_6_*) δ 21.23, 21.29, 24.67 (2C), 25.26, 25.55, 28.46 (2C), 33.51, 36.81, 42.03, 45.29 (2C), 51.96, 59.96, 71.98, 120.49 (2C), 130.15 (2C), 139.26, 148.67, 173.77; HRMS (ESI): *m/z* calcd. for [M+H]^+^: 374.2650; found: 374.2691.

*4-(2-(Dimethylamino)-1-(1-hydroxycyclohexyl)ethyl)phenyl 4-methylcyclohexanecarboxylate* (**1c**). ODV was treated with 4-methylcyclohexane carbonyl chloride (**c**, 1.2 g) to give **1c** (1.1 g, 36.4%) as a white powder; mp 157–159 °C; ^1^H-NMR (DMSO-*d_6_*) δ 0.88 (m, 3H, 4-methylcyclohexane-CH_3_), 0.94–1.75 (m, 19H, 4-methylcyclohexane-CH_2_-, cyclohexane-CH_2_-, 4-methylcyclohexane-CH), 2.04 (m, 1H, CHCOO-), 2.11 (s, 6H, -N(CH_3_)_2_), 2.43–2.90 (m, 3H, -CH_2_-N, Ar-CH), 5.04 (br, 1H, -OH), 6.95 (d, *J* = 8.0 Hz, Ar-H), 7.22 (d, *J* = 8.0 Hz, 2H, Ar-H); ^13^C-NMR (DMSO-*d_6_*) δ 21.10, 21.16, 22.08, 25.40, 28.40 (2C), 31.28, 33.45 (3C), 36.68, 42.13, 45.11 (2C), 51.92, 59.91, 71.87, 120.28 (2C), 129.99 (2C), 139.11, 148.60, 173.70; HRMS (ESI): *m/z* calcd. for [M+H]^+^: 388.2807; found: 388.2850.

*4-(2-(Dimethylamino)-1-(1-hydroxycyclohexyl)ethyl)phenyl 4-isopropylcyclohexanecarboxylate* (**1d**). ODV was treated with 4-isopropylcyclohexane carbonyl chloride (**d**, 1.4 g) to give **1d** (0.7 g, 22.5%) as a white powder; mp 149–152 °C; ^1^H-NMR (DMSO-*d_6_*) δ 0.86 (m, 6H, isopropyl-(CH_3_)_2_), 1.06–2.11 (m, 20H, cyclohexyl-(CH_2_)_5_-, isopropyl-(CH_2_)_4_-, isopropyl-CH, -CH), 2.11 (s, 6H, -N(CH_3_)_2_), 2.45 (m, 1H, CHCOO-), 2.53 (m, 1H, -CH_2_-N), 2.82 (m, 1H, -CH_2_-N), 2.88 (m,1H, Ar-CH), 5.01 (br, 1H, -OH), 6.95 (d, *J* = 8.4 Hz, 2H, Ar-H), 7.22 (d, *J* = 8.4 Hz, 2H, Ar-H); ^13^C-NMR (DMSO-*d_6_*) δ 19.42 (2C), 21.10, 21.17, 25.40, 28.08 (2C), 28.60 (2C), 32.03, 33.50, 36.69, 42.53, 42.60, 45.14 (2C), 51.94, 59.92, 71.88, 120.28 (2C), 129.99 (2C), 139.16, 148.58, 173.69; HRMS (ESI): *m/z* calcd. for [M+H]^+^: 416.3120; found: 416.3147.

*4-(2-(Dimethylamino)-1-(1-hydroxycyclohexyl)ethyl)phenyl cycloheptanecarboxylate* (**1e**). ODV was treated with cycloheptanecarbonyl chloride (**e**, 1.2 g) to give **1e** (1.5 g, 51.8%) as a light yellow powder; mp 110–112 °C; ^1^H-NMR (CDCl_3_) δ 0.95–2.08 (m, 22H, cyclohexane-CH_2_- and cycloheptane-CH_2_-), 2.30 (m, 1H, -CH_2_-N), 2.98 (m, 1H, -CH_2_-N), 2.32 (s, 6H, -N(CH_3_)_2_), 2.73 (m, 1H, CHCOO-), 3.29 (m, 1H, Ar-CH), 6.98 (d, *J* = 8.4 Hz, 2H, Ar-H), 7.13 (d, *J* = 8.4 Hz, 2H, Ar-H); ^13^C-NMR (CDCl_3_) δ 21.30, 21.59, 25.94, 26.32 (2C), 28.31 (2C), 30.75 (2C), 31.20, 38.13, 44.95, 45.47 (2C), 52.00, 61.04, 74.15, 120.93 (2C), 130.06 (2C), 138.00, 149.62, 175.45; HRMS (ESI): *m/z* calcd. for [M+H]^+^: 388.2807; found: 388.2835.

*4-(2-(Dimethylamino)-1-(1-hydroxycyclohexyl)ethyl)phenyl benzoate* (**1f**). ODV was treated with benzoyl chloride (**f**, 1.1 g) to give **1f** (1.4 g, 50.7%) as a white powder; mp 175–178 °C; ^1^H-NMR (DMSO-*d_6_*) δ 0.91–1.61 (m, 10H, cyclohexane-CH_2_-), 2.13 (s, 6H, -N(CH_3_)_2_), 2.88 (m, 2H, -CH_2_-N), 3.29 (m, 1H, Ar-CH), 7.15 (d, *J* = 8.4, 2H, Ar-H), 7.30 (d, *J* = 8.4, 2H, Ar-H), 7.61 (t, *J* = 7.4, 2H, Ar-H), 7.75 (t, *J* = 7.4, 1H, Ar-H), 8.13 (d, *J* =7.4, 2H, Ar-H); ^13^C-NMR (DMSO-*d_6_*) δ 21.23, 21.30, 25.55, 33.62, 36.78, 45.29 (2C), 51.97, 59.92, 71.97, 120.65 (2C), 128.92 (2C), 129.67 (2C), 130.29 (2C), 131.45, 128.61, 139.60, 148.71, 164.56; HRMS (ESI): *m/z* calcd. for [M+H]^+^: 368.2181; found: 368.2224.

*4-(2-(Dimethylamino)-1-(1-hydroxycyclohexyl)ethyl)phenyl furan-3-carboxylate* (**1g**). ODV was treated with furan-3-carbonyl chloride (**g**, 1.0 g) to give **1g** (1.2 g, 42.6%) as a white powder; mp 159–162 °C; ^1^H-NMR (DMSO-*d_6_*) δ 0.97–1.61 (m, 10H, cyclohexane-CH_2_-), 2.12 (s, 6H, -N(CH_3_)_2_), 2.58 (m, 1H, -CH_2_-N), 2.86 (m, 1H, -CH_2_-N), 3.32 (m, 1H, Ar-CH), 4.99 (br, 1H, -OH), 6.80 (m, 1H, furan-H), 7.12 (d, *J* = 8.0, 2H, Ar-H), 7.29 (d, *J* = 8.0, 2H, Ar-H), 7.54 (m, 1H, furan-H), 8.09 (m, 1H, furan-H). ^13^C-NMR (DMSO-*d_6_*) δ 21.11, 21.19, 25.40, 33.68, 36.65, 45.14 (2C), 51.95, 59.87, 71.84, 112.45, 119.74, 120.35 (2C), 130.20 (2C), 139.67, 143.03, 147.92, 148.28, 156.20. HRMS (ESI): *m/z* calcd. for [M+H]^+^: 358.1974; found: 358.1997.

*4-(2-(Dimethylamino)-1-(1-hydroxycyclohexyl)ethyl)phenyl thiophene-3-carboxylate* (**1h**). ODV was treated with thiophene-3-carbonyl chloride (**h**, 1.1 g) to give **1h** (1.5 g, 53.7%) as a white powder; mp 168–172 °C; ^1^H-NMR (DMSO-*d_6_*) δ 1.05–1.64 (m, 10H, cyclohexane-CH_2_-), 2.15 (s, 6H, -N(CH_3_)_2_), 2.57 (m, 1H, -CH_2_-N), 2.87 (m, 1H, -CH_2_-N), 2.94 (m, 1H, Ar-CH), 4.62 (br, 1H, -OH), 7.13 (d, *J* = 8.2, 2H, Ar-H), 7.30 (d, *J* = 8.2, 2H, Ar-H), 7.60 (d, *J* = 4.8, 1H, thiophene-H), 7.69 (m, 1H, thiophene-H), 8.50 (s, 1H, thiophene-H); ^13^C-NMR (DMSO-*d_6_*) δ 20.73, 20.80, 24.92, 33.60, 36.30, 44.61 (2C), 52.01, 59.78, 71.62, 119.73 (2C), 126.98 (2C), 129.63 (2C), 131.91, 133.85, 139.12, 148.27, 159.87; HRMS (ESI): *m/z* calcd. for [M+H]^+^: 374.1745; found: 374.1777.

*4-(2-(Dimethylamino)-1-(1-hydroxycyclohexyl)ethyl)phenyl nicotinate* (**1i**). ODV was treated with nicotinoyl chloride (**i**, 1.1 g) to give **1i** (1.1 g, 38.6%) as a white powder; mp 180–182 °C; ^1^H-NMR (DMSO-*d_6_*) δ 1.04–1.69 (m, 10H, cyclohexane-CH_2_-), 2.68 (s, 6H, -N(CH_3_)_2_), 3.17 (m, 1H, -CH_2_-N), 3.46 (m, 1H, -CH_2_-N), 3.62 (m, 1H, Ar-CH), 5.47 (br, 1H, -OH), 7.33 (d, *J* = 8.3, 2H, Ar-H), 7.48 (d, *J* = 8.3, 2H, Ar-H), 7.71 (m,1H, pyridine-H), 8.51 (m, 1H, pyridine-H), 8.93 (m, 1H, pyridine-H), 9.28 (s, 1H, pyridine-H); ^13^C-NMR (DMSO-*d_6_*) δ 20.95, 21.35, 25.22, 33.54, 36.05, 42.66, 43.30, 50.01, 58.09, 72.04, 121.38 (2C), 124.84, 125.91, 130.74 (2C), 136.94, 139.20, 148.98, 149.33, 152.49, 162.93; HRMS (ESI): *m/z* calcd. for [M+H]^+^: 369.2133; found: 369.2159.

*4-(2-(Dimethylamino)-1-(1-hydroxycyclohexyl)ethyl)phenyl 4-methylbenzoate* (**1j**). ODV was treated with 4-methylbenzoyl chloride (**j**, 1.2 g) to give **1j** (0.8g, 28.2%) as a white powder; mp 159–162 °C; ^1^H-NMR (DMSO-*d_6_*) δ 1.06–1.69 (m, 10H, cyclohexane-CH_2_-), 2.44 (s, 3H, Ar-CH_3_), 2.68 (s, 6H, ‑N(CH_3_)_2_), 3.17 (m, 1H, -CH_2_-N), 3.59 (m, 1H, -CH_2_-N), 3.64 (m, 1H, Ar-CH), 4.66 (br, 1H, -OH), 7.27 (d, *J* = 8.0 Hz, 2H, Ar-H), 7.42-7.47 (dd, *J_1_* =13.2, *J_2_* =8.0, 4H, Ar-H), 8.0 (d, *J* = 8.0 Hz, 2H, Ar-H); ^13^C-NMR (DMSO-*d_6_*) δ 20.95, 21.25, 21.34, 25.22, 33.44, 36.04, 42.71, 43.23, 50.06, 58.10, 72.05, 115.11, 121.49 (2C), 126.18, 129.53 (2C), 129.78 (2C), 130.65, 136.57, 144.53, 149.66, 164.42; HRMS (ESI): *m/z* calcd. for [M+H]^+^: 382.2337; found: 382.2305.

*4-(2-(Dimethylamino)-1-(1-hydroxycyclohexyl)ethyl)phenyl 4-methoxybenzoate* (**1k**). ODV was treated with 4-methoxybenzoyl chloride (**k**, 1.3 g) to give **1k** (0.7 g, 24.3%) as a white powder; mp 133–137 °C; ^1^H-NMR (CDCl_3_) δ 0.90-1.74 (m, 10H, cyclohexane-CH_2_-), 2.57 (m, 1H, -CH_2_-N), 2.72 (s, 6H, -N(CH_3_)_2_), 3.16 (m, 1H, -CH_2_-N), 3.37 (m, 1H, Ar-CH), 3.88 (s, 1H, -OCH_3_), 6.98 (d, *J* = 8.8 Hz, 2H, Ar-H), 7.19 (m, 4H, Ar-H), 8.12 (d, *J* = 8.8 Hz, 2H, Ar-H); ^13^C-NMR (CDCl_3_) δ 21.11, 21.43, 25.24, 31.33, 36.62, 44.05 (2C), 52.69, 55.48, 60.14, 73.59, 113.86 (2C), 121.41, 121.96 (2C), 130.18, 132.22 (2C), 136.67, 150.28, 163.99, 164.77, 165.34; HRMS (ESI): *m/z* calcd. for [M+H]^+^: 398.2287; found: 398.2237.

*4-(2-(Dimethylamino)-1-(1-hydroxycyclohexyl)ethyl)phenyl 3-phenylpropanoate* (**1l***)* ODV was treated with 3-phenylpropanoyl chloride (**l**, 1.3 g) to give **1l** (0.6 g, 19.1%) as a white powder; mp 124–127 °C; ^1^H-NMR (DMSO-*d_6_*) δ 0.95–2.07 (m, 10H, m, 10H, cyclohexane-CH_2_-), 1.96 (s, 6H, -N(CH_3_)_2_), 2.63 (m, 2H, -CH_2_-N), 2.57 (m, 2H, -OCOCH_2_-), 2.70 (m, Ar-CH), 2.82 (m, 2H, Ar-CH_2_-), 6.64 (d, *J* = 8.0, 2H, Ar-H), 6.93 (d, *J* = 8.0, 2H, Ar-H), 7.18-7.29 (m, 5H, Ar-H). ^13^C-NMR (DMSO-*d_6_*) δ 20.84 (2C), 24.63, 30.11, 30.77, 31.00, 35.90, 45.03 (2C), 47.47, 58.84, 85.67, 114.35 (2C), 125.76, 128.00 (2C), 128.05 (2C), 130.19 (3C), 140.47, 155.60, 171.22; HRMS (ESI): *m/z* calcd for [M+H]^+^: 396.2494; found: 396.2509.

*4-(2-(Dimethylamino)-1-(1-hydroxycyclohexyl)ethyl)phenyl cinnamate* (**1m**). ODV was treated with cinnamoyl chloride (**m**, 1.3 g) to give **1m** (0.7 g, 23.8%) as a white powder; mp 118–122 °C; ^1^H-NMR (CDCl_3_) δ 0.87–1.73 (m, 10H, cyclohexane-CH_2_-), 2.38 (s, 6H, -N(CH_3_)_2_), 2.47 (m, 1H, -CH_2_-N), 3.07 (m, 1H, -CH_2_-N), 3.40 (m, 1H, Ar-CH), 5.06 (br, 1H, -OH), 6.88 (d, *J* = 16.0, 1H, -COHC=CH-), 7.09–7.59 (m, 8H, Ar-H), 7.85 (d, *J* = 16.0, 1H, -COHC=CH-); ^13^C-NMR (DMSO-*d_6_*) δ 20.96, 21.35, 25.23, 33.42, 36.05, 42.81, 43.11, 50.09, 58.14, 72.07, 117.23, 121.36 (2C), 128.65 (2C), 128.90, 129.00, 130.43, 130.62, 130.90, 133.80, 136.59, 146.40, 149.49, 164.77; HRMS (ESI): *m/z* calcd. for [M+H]^+^: 394.2337; found: 394.2346.

*4-(2-(Dimethylamino)-1-(1-hydroxycyclohexyl)ethyl)phenyl benzo[d][1,3]dioxole-5-carboxylate* (**1n**). ODV was treated with benzo[d][1,3]dioxole-5-carbonyl chloride (**n**, 1.4 g) to give **1n** (1.5 g, 48.6%) as a white powder; mp 154–157 °C; ^1^H-NMR (DMSO-*d_6_*) δ 0.90-1.66 (m, 10H, cyclohexane-CH_2_-), 2.13 (s, 6H, -N(CH_3_)_2_), 2.54 (m, 1H, -CH_2_-N), 2.87 (m, 1H, -CH_2_-N), 3.32 (m, 1H, Ar-CH), 5.00 (1H, br, -OH), 6.19 (s, 2H, -O-CH_2_-O-), 7.12 (d, *J* = 8.0 Hz, 2H, Ar-H), 7.28 (d, *J* = 8.0 Hz, 2H, Ar-H), 7.55 (s, 1H, Ar-H), 7.75(d,*J* = 1.6 Hz, 1H, Ar-H),7.73 (d, *J* = 1.6Hz, 1H, Ar-H); ^13^C-NMR (CDCl_3_) δ 21.22, 21.49, 25.83, 31.14, 38.04, 45.36 (2C), 51.94, 60.94, 74.05, 101.89, 108.06, 109.79, 121.04 (2C), 123.32, 126.07, 130.06 (2C), 138.12, 147.83. 149.57, 152.12, 164.37; HRMS (ESI): *m/z* calcd. for [M+H]^+^: 412.2079; found: 412.2113.

*4-(2-(Dimethylamino)-1-(1-hydroxycyclohexyl)ethyl)phenyl 2,3-dihydrobenzo[b][1,4]dioxine-6-carboxylate* (**1o**). ODV was treated with 2,3-dihydrobenzo[b][1,4]dioxine-6-carbonyl chloride (**o**, 0.8 g) to give **1o** (1.5 g, 27.5%) as a white powder; mp 159–162 °C; ^1^H-NMR (CDCl_3_) δ 1.02–1.72 (m, 10H, cyclohexane-CH_2_-), 2.37 (s, 6H, -N(CH_3_)_2_), 2.42(m, 1H, -CH_2_-N), 3.06(m, 1H, -CH_2_-N), 3.37(m, 1H, Ar-CH), 4.31(m, 2H, -O-(CH_2_)_2_-O-), 4.33 (m, 2H, -O-(CH_2_)_2_-O-), 7.18(d, *J* = 8.0 Hz, 2H, Ar-H), 7.11(d, *J* = 8.0 Hz, 2H, Ar-H), 6.95 (d, *J* = 8.8 Hz, 1H, Ar-H), 7.71(d, *J* = 8.8 Hz, 1H, Ar-H), 7.72 (s, 1H, Ar-H); ^13^C-NMR (CDCl_3_) δ 21.29, 21.51, 25.83, 31.19, 37.98, 45.31 (2C), 52.06, 60.98, 64.06, 64.66, 74.08, 117.31, 119.53, 121.18 (2C), 122.60, 124.06, 130.09 (2C), 138.01, 143.28, 148.37, 149.71, 164.58; HRMS (ESI): *m/z* calcd. for [M+H]^+^: 426.2236; found: 426.2269.

### 3.4. PK Studies in Rat

Mature normal class Wistar rats (aged 4–6 weeks, weight 200 ± 20 g) were obtained from the Animal Research Institute, Jilin University. Animals were housed under controlled temperature and humidity conditions and allowed free access to food and water. After a 12 h fast, rats were administered a single dose of 0.02 mmol/kg drug by oral gavage. Blood samples (250 μL) were collected into heparinized. Eppendorf tubes before dosing and at 0.167, 0.33, 0.5, 1, 1.5, 2.0, 3.0, 4.0, 6.0, 8.0, 12.0 and 24.0 h after dosing. After centrifugation at 3500 rpm for 5 min, 100 μL plasma was removed and stored at −80 °C until analysis by LC-MS/MS. The system consisted of an Agilent 1100 Series HPLC (Agilent Technologies, Palo Alto, CA, USA) connected to a QTRAP 2000TM mass spectrometer (Applied Biosystems Sciex, Concord, ON, Canada), equipped with an ESI source operated in the positive ion mode. HPLC separation was performed on an Agilent SB-AQ column (150 × 4.6 mm, 5 μm, Agilent Technologies) maintained at 40 °C using a mobile phase of methanol:10 mM ammonium acetate (85:15, v:v). Applied Biosystems/MDS SCIEX Analyst software (Version 1.3.2) was used for data acquisition and processing. PK parameters were estimated by a non-compartmental method using DAS 3.0 software package (Mathematical Pharmacology Professional Committee of China, Shanghai, China). 

### 3.5. Brain Uptake Studies in Rat

After a 12 h fast, rats were administered a single dose of 0.06 mmol/kg by oral gavage. At 0.25, 0.5, 1, 2, and 4 h after dosing, rats were sacrificed and perfused with 40 mL PBS through the left ventricle. The brain was removed and the hypothalamus was dissected out. The hypothalamus and total brain were then placed in 1 mL and 2 mL respectively of ice-cold methanol:water (1:1, v:v) and maintained on ice until homogenization using a Tempest Virtishear tissue homogenizer (Virtis, Apache Junction, AZ, USA) for about 45 s followed by sonication using a Heat Systems Ultrasonic sonicator for 1 min. After centrifugation at 3,500 rpm for 5 min, the supernatant was separated and stored at −80 °C until analysis.

### 3.6. PK Studies in Beagle Dog

Beagle dogs (10 ± 2 kg, n = 6) were obtained from the Chengdu Dashuo Biological Technology Co., Ltd (Sichuan, China). After a 12 h fast, dogs were administered a single dose of 0.007 mmol/kg of either compound **1n** or ODV by oral gavage on two occasions separated by a one week washout period. Blood samples (1 mL) were collected into heparinized Eppendorf tubes before dosing and at 0.083, 0.167, 0.33, 0.5, 0.75, 1.0, 1.5, 2.0, 3.0, 5.0, 7.0, 11.0 and 24.0 h after dosing. After centrifugation at 3500 rpm for 5 min, plasma was collected and 100 µL aliquots stored at −80 °C until analysis.

## 4. Conclusions

In this study, a series of 15 phenolic esters of ODV **1a**–**o** were synthesized and their PK evaluated in rat in comparison with ODV itself. The four compounds with the highest relative bioavailabilities (compounds **1c**, **1e**, **1n**, and **1o**) were selected for brain uptake studies in rat in which compound **1n** exhibited the highest levels in both hypothalamus and total brain. Finally the PK of **1n** was compared with ODV in beagle dogs where it again demonstrated a relative bioavailability increase similar to that in rat. On the basis of these results, the newly synthesized ODV piperonylic acid ester **1n** is the most promising candidate for development as an ODV prodrug.
